# Reporting perioperative complications of radical cystectomy: the influence of using standard methodology based on ICARUS and EAU quality criteria

**DOI:** 10.1186/s12957-023-02943-9

**Published:** 2023-02-23

**Authors:** Naci Burak Cinar, Hasan Yilmaz, Ibrahim Erkut Avci, Kutlucan Cakmak, Kerem Teke, Ozdal Dillioglugil

**Affiliations:** Department of Urology, Kocaeli University School of Medicine, 41380 Izmit, Kocaeli Turkey

**Keywords:** Charlson comorbidity index, Icarus criteria, Inflammatory-nutritional markers, Intraoperative complication, Martin criteria, Open radical cystectomy, Systemic inflammatory response index

## Abstract

**Purpose:**

We aimed to evaluate perioperative complications of radical cystectomy (RC) by using standardized methodology. Additionally, we identified independent risk factors associated with perioperative complications.

**Materials and methods:**

We retrospectively analyzed 30-day and 90-day perioperative complications of 211 consecutive RC patients. The intraoperative and postoperative complications were defined according to Clavien-Dindo classification (CDC) and reported based on the ICARUS criteria, Martin, and EAU quality criteria. Age-adjusted Charlson comorbidity index (ACCI), systemic inflammatory response index (SIRI), body mass index (BMI) ≥ 25 kg/m^2^, and neoadjuvant chemotherapy (NAC) were also evaluated. Multivariable regression models according to severe (*CDC* ≥ IIIb grade) complications were tested.

**Results:**

Overall, 88.6% (187/211) patients experienced at least one intraoperative complication. Bleeding during cystectomy was the most common complication observed (81.5% [172/211]). Severe intraoperative complications (EAUiaiC grade > 2) were recorded in 8 patients. Overall, 521 postoperative complications were recorded. Overall, 69.6% of the patients experienced complications. Thirty-nine patients suffered from most severe (*CDC* ≥ IIIb grade) complications. ACCI (*OR*: 1.492 [1.144–1.947], *p* = 0.003), SIRI (*OR*: 1.279 [1.029–1.575], *p* = 0.031), BMI (*OR*: 3.62 [1.58–8.29], *p* = 0.002), and NAC (*OR*: 0.342 [0.133–0.880], *p* = 0.025) were significant independent predictive factors for 90-day most severe complications (*CDC* ≥ IIIb grade).

**Conclusions:**

RC complications were reported within a standardized manner, concordant with the ICARUS and Martin criteria and EAU guideline recommendations. Complication reporting seems to be improved with the use of standard methodology. Our results showed that ACCI, SIRI, and BMI ≥ 25 kg/m^2^ and the absence of NAC were significant predictive factors for most severe complications.

**Supplementary Information:**

The online version contains supplementary material available at 10.1186/s12957-023-02943-9.

## Introduction

Radical cystectomy (RC) is one of the most difficult and invasive surgical procedures in urology. The surgery involves both gastrointestinal and urinary systems after radical resection of the bladder. Hence, RC has higher perioperative complication rates. The reported perioperative complication rates vary from 19% up to 99% within the first 90 postoperative days [1–6]. This big discrepancy may be explained by lack of standardized definitions of perioperative complications. Well-organized data collection with standard assessment is an important aspect of reporting outcomes. In 2002, Martin et al. proposed ten criteria for reporting complications following surgery to achieve a uniform and standardized approach [7]. This concept was adopted by the European Association of Urology (EAU) in their guideline for complication reporting [8]. Additionally, recently, the EAU panel released the Intraoperative Complications Assessment and Reporting with Universal Standards (ICARUS) criteria for reporting intraoperative adverse events [9]. However, in our knowledge, there is no study in the literature that reported complications accordingly fulfilled EAU intraoperative and postoperative quality criteria.

In this study, first, we aimed to evaluate the intraoperative and the 30-day and 90-day postoperative mortality and complications of open RC by using a standardized reporting methodology according to EAU guidelines. Second, we aimed to identify the associated independent risk factors for postoperative complications.

## Materials and methods

### Patient population

We conducted a retrospective analysis of perioperative complications operated in our tertiary referral institution. We identified 251 consecutive individuals who had undergone open standard RC, bilateral extended pelvic lymph node dissection, and urinary diversion (ileal loop or Studer pouch) for bladder cancer between 2009 and 2021. All patients had at least 90-day follow-up or died within 90 days. Patients who underwent an additional nephroureterectomy (*n* = 19), urinary diversion with ureterocolostomy (*n* = 3), and pelvic exenteration procedure (*n* = 2); who had previous radiotherapy (*n* = 6) and non-urothelial carcinoma (*n* = 5); and those who had two or more procedure simultaneously (*n* = 5) were excluded (Supplementary Fig. 1). The remaining 211 patients met the inclusion criteria. Surgery was performed by five surgeons, whereas two of them were trained during the study period. For perioperative care after RC, a protocol similar to the modified ERAS has been used in our clinic since 2015. In 2019, our protocol was revised to be more compatible with ERAS. The patients with full compliance to ERAS was 22.2% (47/211).

### Data acquisition

In order to obtain a detailed perioperative 90-day complication follow-up, two urology residents (B. C., K. C.) screened our medical records for inpatient, outpatient, and emergency clinics. Patient demographics, all intraoperative and postoperative 30-day and 90-day complications and causes of readmission, reoperation, and mortality were noted. Comorbidities were also assessed with age-adjusted Charlson comorbidity index (ACCI) [10].

We calculated the following previously well-known inflammatory-nutritional markers reported in the literature, based on the blood tests on the day before surgery: (1) the platelet-to-lymphocyte ratio (PLR) [11]; (2) albumin-to-globulin ratio (AGR) [12]; (3) the systemic inflammatory response index (SIRI) (the product of neutrophils and monocytes, divided by lymphocyte count) [13–17]; and (4) the prognostic nutritional index (PNI) [(10 × blood albumin count) + 0.005 × blood lymphocyte count)] [18].

### Assessment of complications

In order to adhere to the EAU guidelines for reporting and grading complications, the intraoperative complications were defined and reported based on ICARUS criteria [9] and the postoperative complications based on EAU proposal [8] and also the Martin criteria [7]. Intraoperative complications were graded according to EAU intraoperative adverse incident classification (EAUiaiC) [19]. Each postoperative complication was graded according to a modified version of the CDC [20].

The study was approved by the local ethics committee (approval number: KÜ GOKAEK-2022/05.06). The informed and signed consent has been obtained from all patients prior to surgery.

### Statistical analysis

Statistical analyses were performed using SPSS Statistics 21 (IBM, Armonk, NY, USA). A *p* < 0.05 indicated statistical significance. The independent Student *t*-test and Mann-Whitney *U* were used for parametric and nonparametric values. Chi-square test was used for categorical values. Patient demographics were stratified and compared according to ACCI (< 6 vs. ≥ 6).

Univariable and multivariable logistic regression models were used to predict the most severe complication *CDC* ≥ grade IIIb (separately for 30 days and 90 days). All factors that could potentially influence the most severe complications were tested with simple logistic regression analyses. BMI was categorized as dichotomous variable according to the presence of obesity (< 25 kg/m^2^/≥ 25 kg/m^2^).

Receiver operating characteristic (ROC) curve analyses were used to assess the true-positive (sensitivity) and false-positive rates (1- specificity) of SIRI and ACCI with reference to the most severe complication binary outcome. ROC curve analyses were performed with the MedCalc 20.106 trial version. The areas under the curve (AUC) were calculated. The value with the highest sensitivity and specificity was selected as the cutoff value (Youden index J).

## Results

Two-hundred eleven patients are included in the analyses. Demographics according to ACCI groups are given in Table 1. About 45% (94/211) of patients had *ACCI* ≥ 6 comorbidity. These patients had higher median age and incidence of prior pelvic surgery and had lower e-GFR and operation time; they were less smokers (*p* < 0.05). Statistically significant difference was found between ACCI groups according to pathological stages and characteristics.

**Table 1:** Demographics accordingly ACCI groups

	Overall	***ACCI*** < 6	***ACCI*** ≥ 6	***p***
**Clinical preoperative characteristics**				
Number of patients	211	117	94	
Age (y), median (IQR)	65 (60–70)	61 (56.5–67)	69.5 (64–73)	**< 0.001**^c^
Gender: male, *n* (%)	181 (85.8)	100 (85.5)	81 (86.2)	0.885^a^
BMI (kg/m^2^), median (IQR)	25.4 (23.8–27.6)	25.4 (23.7–27.5)	25.4 (23.8–27.7)	0.850^b^
Hemoglobin (g/dl), median (IQR)	12.1 (11–13.4)	12.3 (11.2–13.7)	11.6 (10.6–12.9)	**0.015**^b^
e-GFR before cystectomy (ml/min/1.73 m^2^), median (IQR)	79 (52–92)	81 (63.5–96.7)	67.9 (45.0–87.0)	**< 0.001**^c^
Hydronephrosis, *n* (%)	62 (29.4)	37 (31.6)	25 (26.6)	0.451^a^
Smoking, *n* (%)	168 (79.6)	99 (84.6)	69 (73.4)	**0.044**^a^
Prior pelvic or abdominal surgery, *n* (%)	53 (25.1)	23 (19.7)	30 (31.9)	**0.041**^a^
Intravesical treatment, *n* (%)	34 (16.2)	18 (15.2)	16 (17.0)	0.899^a^
Prior systemic chemotherapy, *n* (%)	38 (18)	21 (17.9)	17 (18.1)	0.980^a^
**Surgical characteristics**				
Operative time (min), median (IQR)	420 (370–480)	420 (390–497.5)	420 (360–480)	**0.031**^c^
Intraoperative blood loss (ml), median (IQR)	1100 (700–1725)	1100 (800–1675)	1100 (650–1925)	0.840^c^
Length of stay (d), median (IQR)	12 (11–15)	12 (11–14)	12 (11–15)	0.559^c ^
Mortality, *n* (%)	126 (59.7)	64 (54.7)	62 (66)	0.098^c^
**Clinical stage**				0.436^c^
cT1, *n* (%)	13 (6.2)	5 (4.3)	8 (8.5)	
cT2, *n* (%)	106 (50.2)	59 (50.4)	47 (50)	
cT3, *n* (%)	65 (30.8)	36 (30.8)	29 (30.8)	
cT4, *n* (%)	27 (12.8)	17 (14.5)	10 (10.6)	
**Cystectomy characteristics**				
pT stage				0.052^c^
pT0, *n* (%)	12 (5.7)	7 (6)	5 (5.3)	
pTa, *n* (%)	6 (2.9)	2 (1.7)	4 (4.3)	
pT1, *n* (%)	18 (8.5)	8 (6.8)	10 (10.6)	
pT2, *n* (%)	54 (25.6)	35 (29.9)	19 (20.2)	
pT3, *n* (%)	69 (32.7)	45 (38.5)	24 (25.5)	
pT4, *n* (%)	50 (23.7)	20 (17.1)	30 (31.9)	
CIS, *n* (%)	2 (0.9)	0	2 (0.9)	
pN stage				0.530^a^
pN0, *n* (%)	133 (63)	71 (60.7)	62 (65.9)	
pN+, *n* (%)	78 (37)	46 (39.3)	32 (34.1)	
Number of lymph nodes resected, median (IQR)	19 (14-27)	19 (15-28)	18.5 (13-25.5)	0.234^c^
Variant histology, *n* (%)	41 (19.4)	22 (18.8)	19 (20.2)	0.797^a^
Lymphovascular invasion, *n* (%)	90 (42.7)	51 (43.6)	39 (41.5)	0.759^a^
Presence of CIS, *n* (%)	61 (26.8)	28 (23.9)	29 (30.9)	0.261^a^
Positive surgical margin, *n* (%)	13 (6.2)	10 (8.5)	3 (3.2)	0.104^a^
Urinary diversion type				**0.023**^a^
Continent (Studer), *n* (%)	10 (4.7)	9 (7.7)	1 (1.1)	
Incontinent (ileal loop), *n* (%)	201 (95.3)	108 (92.3)	93 (98.9)	

*Abbreviations: ACCI* age-adjusted Charlson comorbidity index, *CIS* carcinoma in situ, *IQR* interquartile range

The bold *p*-values indicate a *p* < 0.05, which is a statistically significant difference

^a^Chi-square test. ^b^Student’s *t*-test. ^c^Mann-Whitney *U*-test

Reporting of the intraoperative complications according to ICARUS criteria are given in Table 2. Overall, 196 intraoperative complications were recorded in 211 patients. Overall, 88.6% (187/211) patients experienced at least one intraoperative complication. Bleeding during cystectomy caused transfusion was the most common complication observed (81.5% [172/211]). Severe intraoperative complications (EAUiaiC grade > 2) were recorded in 8 patients. Two complications were observed due to malfunction of the surgical instruments (bowel stapler).

**Table 2:** Definition of intraoperative complications according to ICARUS criteria

Intraoperative adverse events (iAEs)	Definition of the event	The grade of the iAEs	iAEs related to	No events	Preexisting medical conditions, atypical anatomical variants, and malfunctioning surgical instruments	iAEs recures conversion	Surgical step	The timing of iAE assessment	The management of iAEs	The clinical consequences of a given iAE in the postoperative course
**Bleeding requiring transfusion**	External iliac artery injury	3	Surgery	1	No	No	Pelvic lymphadenectomy	At the time of operation	Suturing with 4.0 Prolene® by cardiovascular surgery	Drainage and hemogram monitoring
	External iliac vein injury	3	Surgery	2	No	No	Pelvic lymphadenectomy	at the time of operation	Suturing with 4.0 Prolene® by cardiovascular surgery	Drainage and hemogram monitoring
	Bleeding superior vagina	3	Surgery	1	No	No	Hysterectomy	At the time of operation	Repair with 3.0 Vicryl® by obstetric surgeon	Drainage and hemogram monitoring
	Bleeding during cystectomy	1	Surgery	172	No	No	Cystectomy	At the time of operation	Blood transfusion and bleeding control	Drainage and hemogram monitoring
**Anemia without bleeding**	Blood transfusion	1	Anesthesiology	7	Having preoperative anemia	No	Cystectomy	At the time of operation	Blood transfusion and hydration	The patient’s hemodynamic was followed
**Bowel injury**	Rectal injury	2	Surgery	3	No	No	Prostatectomy	At the time of operation	Repair with 2.0 silk and Vicryl® by general surgeon	Delayed initiation of oral feeding
		3	Surgery	1	Due to tumor invasion into the rectum	No	Prostatectomy	At the time of operation	The rectum was resected with a linear stapler. Colostomy was performed by GS	Delayed initiation of oral feeding
	Ileal anastomosis leak	4b	Malfunctioning surgical instruments	1	Linear stapler	No	Ileal anastomosis	Post operation	The anastomosis leak was repaired by reoperation	NPO total parenteral nutrition was started
	Appendix injury	4a	Surgery	1	Intra-abdominal adhesions	No	Ileal anastomosis	At the time of operation	Appendectomy by general surgeon	No
	Caecum injury	2	Surgery	1	No	No	During surgical exclusion	At the time of operation	Repair with 2.0 silk by general surgeon	Delayed initiation of oral regimen
	Ileum injury	4b	Malfunctioning surgical instruments	1	Cautery injury	No	Ileal anastomosis	At the time of operation	Side-to-side anastomosis by general surgeon	NPO total parenteral nutrition was started
**Urinary organ injury**	Bladder injury	2	Surgery	2	Tumor invasion into surrounding tissues	No	Bladder excision	At the time of operation	Bladder repair with 3.0 Vicryl®	Distilled water was added during the operation
	Ureter injury	2	Surgery	1	No	No	Ureter implantation	At the time of operation	Anastomosis was done again	Prolonged ureteral stent
	Urethral fragmentation	2	Surgery	1	No	No	Urethrectomy	At the time of operation	Total urethrectomy	
**Nerve injury**	Obturatory nerve damage	2	Surgery	1	No	No	Prostatectomy	At the time of operation	Obturatory nerve repair with 6.0 Vicryl®	Lower extremity adduction movement limitation
**Overall**				196						

*Abbreviations: NPO* nothing per oral, Vicryl®; Polyglactin, Prolene®; Polypropylene

**Fig. 1:**
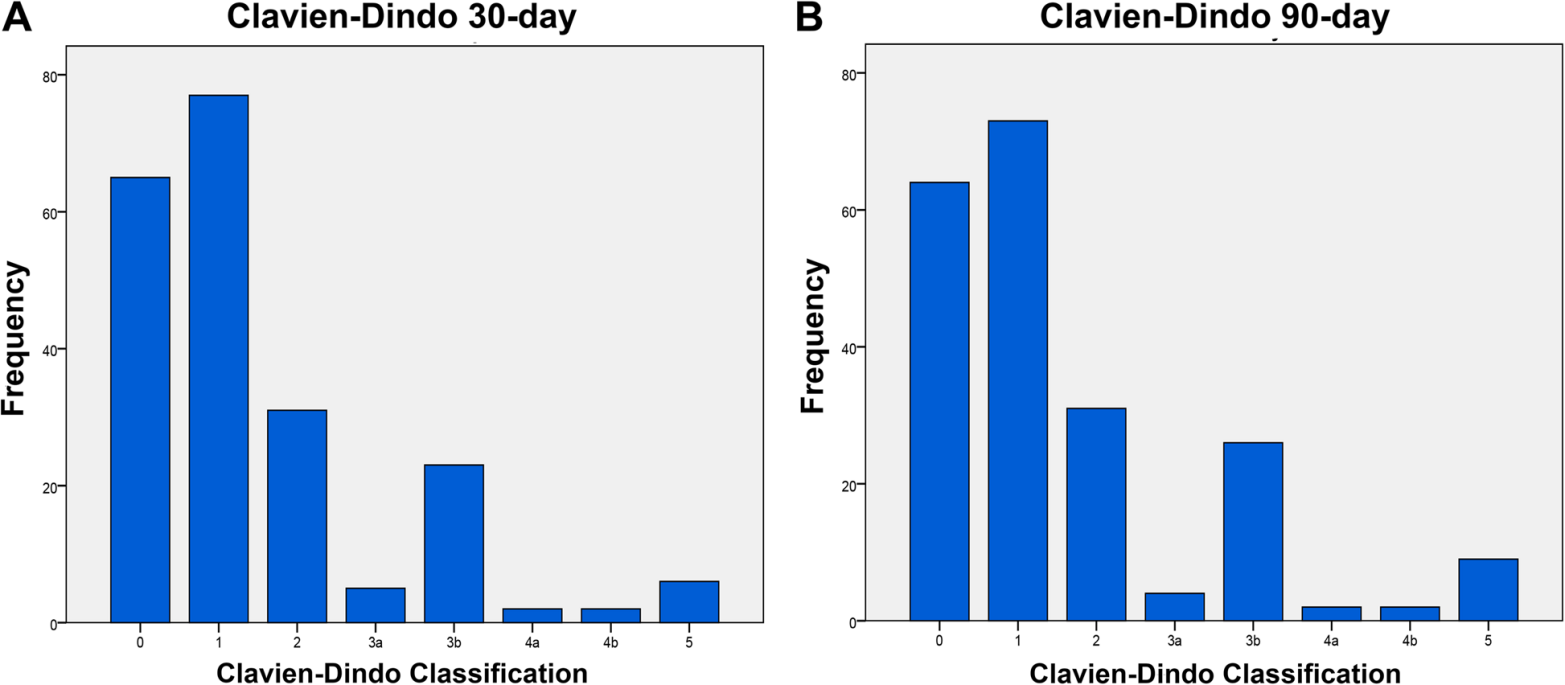
Distribution of complications stratified by CDC. **A** 30-day complications. **B** 90-day complications

Detailed information about type, severity, and distribution of postoperative complications across different organ systems are given in Table 3. Overall, 521 postoperative complications were recorded in 211 patients (30 days *n* = 467 and 90 days *n* = 521). Overall, 69.6% (147/211) patients experienced at least one or more complications. Overall, 45.0% (95/211) of patients had > 1 complications. Gastrointestinal (26.9% at 30 days and 27.8% at 90 days) complications were most commonly observed, while thromboembolic complications were the least common (1.4% at 30 days and 1.7% at 90 days). Thirty-three (15.6%) and 39 (18.4%) patients suffered from most severe complications, requiring an intervention under general anesthesia at 30 days and 90 days, respectively. The distribution of complications according to the CDC is given in Fig. 1.

**Table 3:** Detailed information about type, severity, and distribution of complications across different organ systems

			POD	
Complications	CDC grade	Management	0–30	0–90
			*n* (%)	*n* (%)
**Gastrointestinal**			**126 (26.9)**	**145 (27.8)**
Ileus (paralytic)	1	Conservative	19 (4)	20 (3.8)
	2	Replacement of nasogastric tube	12 (2.5)	17 (3.2)
	3b	Laparotomy	6 (1.2)	13 (2.4)
Small bowel obstruction (mechanical)	3b	Laparotomy	2 (0.4)	2 (0.3)
Constipation	1	Conservative	14 (2.9)	15 (2.8)
GIS bleeding	2	Blood transfusion	6 (1.2)	6 (1.1)
Emesis	1	Conservative	53 (11.3)	57 (10.9)
Anastomotic bowel leakage	3b	Laparotomy	4 (0.8)	4 (0.7)
Diarrhea	1	Conservative	10 (2.1)	11 (2.1)
**Infectious**			**56 (11.9)**	**65 (12.4)**
Fever of unknown	2	Conservative	26 (5.5)	28 (5.3)
Lower urinary tract infection	2	Antibiotic treatment	16 (3.4)	17 (3.2)
Abscess	2	Antibiotic treatment	4 (0.8)	5 (0.9)
	3a	Incision and drainage	2 (0.4)	4 (0.7)
	3b	Incision and drainage (under general anesthesia)	1 (0.2)	3 (0.5)
Sepsis	2	Antibiotic treatment	2 (0.4)	2 (0.3)
	4b	Multiorgan dysfunction	3 (0.6)	4 (0.7)
Pyelonephritis	2	Antibiotic treatment	1 (0.2)	1 (0.1)
Cholecystitis	3b	Cholecystectomy	1 (0.2)	1 (0.1)
**Wound**			**24 (5.1)**	**26 (4.9)**
Wound seroma	1	Conservative	6 (1.2)	7 (1.3)
Wound dehiscence (fascia intact)	2	Antibiotic treatment and saturation under local anesthesia	6 (1.2)	7 (1.3)
Evisceration	3b	Secondary surgical closure	12 (2.5)	12 (2.2)
**Genitourinary**			**52 (10.9)**	**63 (12)**
Acute kidney injury	1	Conservative	40 (8.4)	50 (9.4)
	4a	Dialysis	5 (1)	5 (0.9)
Urinary leak/urinoma	3b	Ureteral reimplantation	1 (0.2)	1 (0.1)
Incisional hernia	3b	Laparotomy and surgical revision	3 (0.6)	3 (0.5)
Urostomy ischemia	1	Conservative	0	1(0.1)
	3b	Laparotomy	1 (0.2)	1 (0.1)
Hematuria	1	Conservative	2 (0.4)	2 (0.3)
**Cardiac**			**20 (4.2)**	**20 (3.8)**
Arrythmia	2	Conservative, medical treatments	2 (0.4)	2 (0.3)
Myocardial infarction	4a	Coronary angiography stent	2 (0.4)	2 (0.3)
Hypertension	2	Medical treatment	2 (0.4)	2 (0.3)
Angina	1	Conservative	2 (0.4)	2 (0.3)
Hypotension	2	Medical treatment	12 (2.5)	12 (2.3)
**Pulmonary**			**23 (4.9)**	**23 (4.4)**
Pneumonia	2	Antibiotic	4 (0.8)	4 (0.7)
Dyspnea	1	Oxygen treatment	19 (4)	19 (3.6)
**Bleeding**			**23 (4.9)**	**28 (5.3)**
Anemia req. transfusion	2	Blood transfusion	23 (4.9)	28 (5.3)
**Thromboembolic**			**7 (1.4)**	**9 (1.7)**
Deep vein thrombosis	2	Anticoagulants	6 (1.2)	8 (1.5)
Pulmonary emboli	2	Anticoagulants	1 (0.2)	1 (0.1)
**Neurological**			**19 (4)**	**20 (3.8)**
CVA-TIA	2	Antiplatelet/anticoagulant	10 (2.1)	11 (2.1)
Delirium	2	Antipsychotic	9 (1.9)	9 (1.7)
**Miscellaneous**			**111 (23.7)**	**113 (21.6)**
Prolonged lymphatic drainage (> 11 days)	1	Conservative — clinical observation	111 (23.7)	113 (21.6)
**Death**			**6 (1.2)**	**9 (1.7)**
Sepsis	5		1 (0.2)	3 (0.5)
Acute kidney failure	5		0	1 (0.1)
Ventricular fibrillation	5		1 (0.2)	1 (0.1)
Disseminated Intravascular coagulation	5		1 (0.2)	1 (0.1)
Electrolyte disorders	5		2 (0.4)	2 (0.3)
Aspiration pneumonia	5		1 (0.2)	1 (0.1)
**Total**			**467 (100)**	**521 (100)**

*Abbreviations: POD *postoperative day, *CDC *Clavien-Dindo classification, *TIA* transient ischemic attack

A total of 9 patients (4.2%) died of various non-cancer-related causes within 90 days of surgery, and these are specified in Table 3. Reasons for reoperations and readmissions are given in Supplementary Table 1. Reoperations related to the primary surgery were performed in 20 (9.4%) patients and were most commonly ileus (*n* = 19) related (Table 3). Thirty-five patients were readmitted to the hospital in 30 days and 42 patients in 90 days. Gastrointestinal causes are most common (Supplementary Table 1). The compliance of our postoperative complication recording with EAU quality criteria is given in Supplementary Table 2.

In the logistic regression analyses, in addition to preoperative clinical inflammatory-nutritional indexes, which are frequently encountered in the literature, we also evaluated other operative and preoperative factors for the prediction of most severe (*CDC* ≥ grade IIIb) complications (Table 4). For 30-day complications, ACCI (*p* < 0.001) and *BMI* ≥ 25 kg/m^2^ were significant independent factors in multivariable analysis, while at 90-day complications, the presence of neoadjuvant chemotherapy (NAC) and SIRI was also found to be independent predictive factors in addition to ACCI and *BMI* ≥ 25 kg/m^2^. It is obviously seen from the low OR in Table 4 (*OR* 0.342) that the absence of NAC increases complication rates. Additionally, SIRI was significantly higher in patients with pT4 disease and positive surgical margins (median *SIRI* 1.90 vs. 1.66; *p* = 0.037).

**Table 4:** Multivariable logistic regression analyses of association between possible predictive factors and the presence of most severe *complication (CDC ≥ 3b)*

	30 days				90 days			
	*Univariable*		*Multivariable*		*Univariable*		*Multivariable*	
	***P***	OR (95% ***CI***)	***P***	OR (95% ***CI***)	***P***	OR (95% ***CI***)	***P***	OR (95% ***CI***)
**Age**	0.345	1.02 (0.98–1.07)			0.183	1.03 (0.99–1.08)		
**BMI ≥ 25 kg/m**^**2**^	0.019	2.51 (1.16–5.43)	**0.020**	2.66 (1.17–6.05)	**0.003**	3.06 (1.47–6.36)	**0.002**	3.62 (1.58–8.29)
**Preoperative Hb**	0.779	0.97 (0.79–1.19)			0.333	0.91 (0.75–1.10)		
**Preoperative albumin**	0.910	0.97 (0.54–1.74)			0.742	0.91 (0.53–1.57)		
**Albumin/total protein**	0.967	1.07 (0.04–30.15)			0.982	0.97 (0.04–22.27)		
**e-GFR**	**0.039**	0.99 (0.97–1.00)	0.408	0.99 (0.97–1.01)	**0.003**	0.98 (0.97–0.99)	0.296	0.99 (0.97–1.00)
**Presence of hydronephrosis**	0.173	0.58 (0.27–1.26)			0.324	0.69 (0.33–1.44)		
**Prior abdominal surgery**	0.756	0.88 (0.38–2.03)			0.934	0.97 (0.44–2.15)		
**Prior systemic chemotherapy**	0.136	0.52 (0.22–1.23)			**0.025**	0.40 (0.18–0.89)	**0.026**	0.34 (0.13–0.88)
**Operation time**	0.778	1.00 (0.99–1.01)			0.492	0.99 (0.99–1.00)		
**ACCI**	**< 0.001**	1.56 (1.22–1.98)	**0.002**	1.49 (1.15–1.91)	**< 0.001**	1.55 (1.24–1.93)	**0.003**	1.49 (1.14–1.94)
**SIRI**	**0.012**	1.21 (1.04–1.41)	0.347	1.09 (0.90–1.34)	**< 0.001**	1.32 (1.14–1.55)	**0.031**	1.27 (1.02–1.57)
**PNI**	0.910	0.10 (0.94–1.06)			0.687	0.99 (0.94–1.04)		
**AGR**	0.924	1.02 (0.73–1.41)			0.827	0.97 (0.70–1.33)		
**PLR**	**0.039**	1.01 (1.00–1.01)	0.189	1.00 (0.99–1.01)	**0.020**	1.01 (1.00–1.01)	0.392	1.00 (0.99–1.01)

*Abbreviations: CDC *Clavien-Dindo classification, *BMI* body mass index, *e-GFR* estimated glomerular filtration rate, *SIRI* systemic inflammatory response index, *PNI* prognostic nutritional index, *AGR* albumin-to-globulin ratio, *PLR* platelet-to-lymphocyte ratio

The bold *p*-values indicate a *p* < 0.05, which is a statistically significant difference

**Fig. 2:**
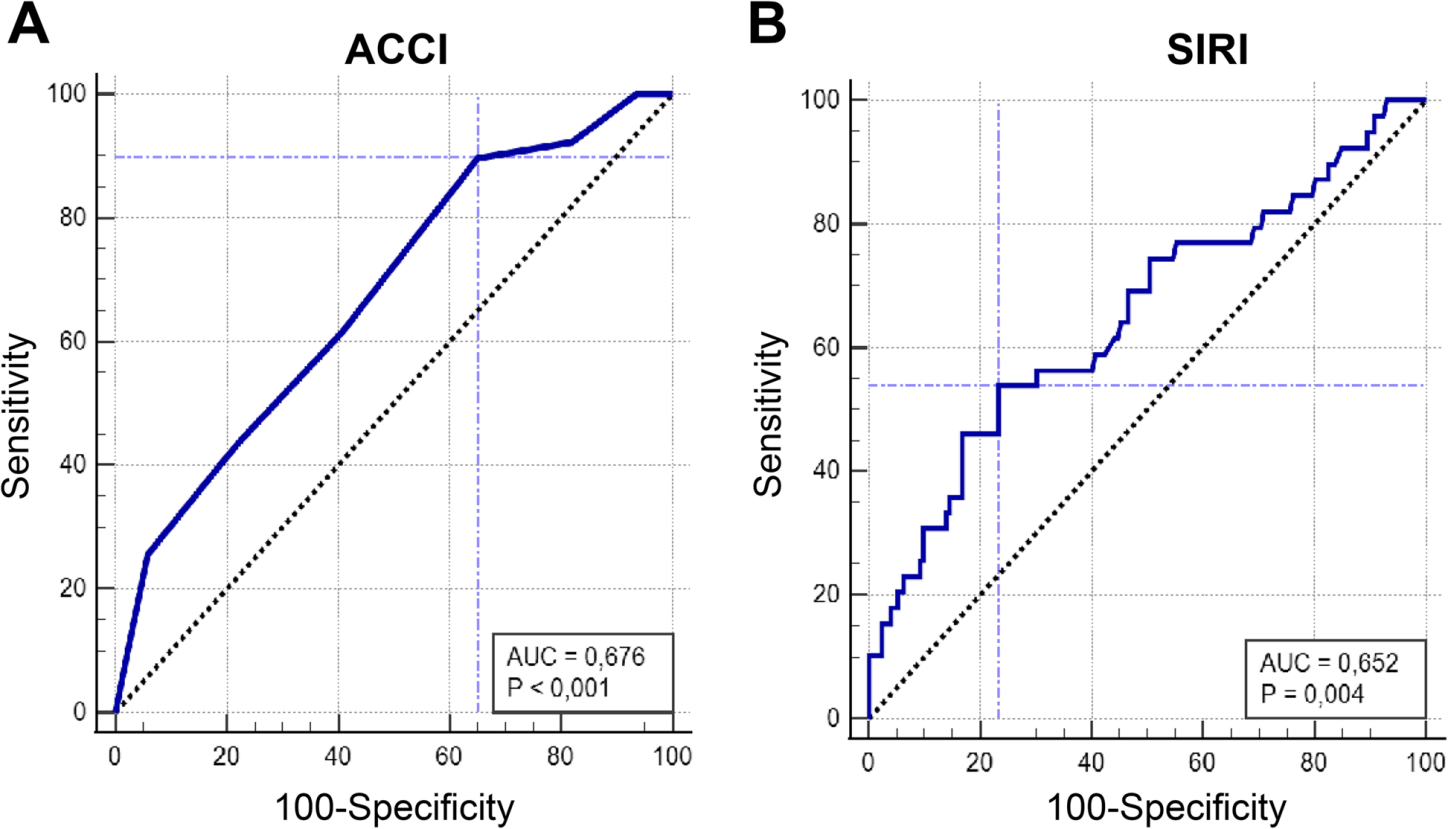
ROC curves: true positive (sensitivity) and false positive (100-specificity) values of ACCI (AUC 0.676; *p*<0.001) and SIRI (AUC 0.649; *p*=0.009) according to 90-day most severe complications (CDC≥IIIb)

We further analyzed SIRI and ACCI in ROC curve analyses to determine a cutoff value to predict 90-day most severe *CDC* ≥ grade IIIb complication. We found SIRI cutoff value of > 2.35 (53.8% sensitivity, 76.7% specificity) and ACCI cutoff value of > 4 (89.7% sensitivity, 34.9% specificity) for accurate prediction of *CDC* ≥ grade IIIb complications (Fig. 2).

## Discussion

RC is associated with high risk of complications. Especially, RC candidates tend to be elderly and have multiple comorbidities. In the current study, we reported meticulous and comprehensive evaluation of perioperative complications. Additionally, we paid attention to meet both the ICARUS criteria [9], Martin criteria [7], and the EAU guideline recommendations for complications [8]. Studies that reported complications in a standard manner are lacking in the literature. Maibom et al. conducted a systematic review of the prevalence of short-term (< 90 days) morbidity and mortality following RC [6]. They selected 66 out of 1957 articles that met the inclusion criteria. Only three of them are compatible with the 10/10 Martin criteria. The median number fulfilling the Martin criteria was 6 (range 2–10).

As a result of more detailed evaluation and reporting of the complications, reported number of complications in the studies is also increasing. In 2002, Meller et al. claimed that RC complications occur in 19% of patients [5]. Contrarily, Vetterlein et al. suggested that 99% of their patients experienced at least one complication [2]. The definition of surgical complication is generally defined as any deviation from the ideal postoperative course that is not inherent in the procedure [8]. However, in defining surgical complications, subjectivity cannot always be avoided. This could also be the reason for this inconsistency. Interestingly, Vetterlein et al. reported urinary tract infection in 62% of the patients (although they did not specify whether the infection was detected by urine culture or only by urinalysis), hematuria in 67% (not specified whether microscopic or macroscopic; probably most of them is not a deviation from an ideal course), and new-onset hydronephrosis in 41% (not specified) [2]. On the other hand, Haas et al. reported 85.7% complications for RC [1]. However, the largest part of this was related to intraoperative blood transfusion (21.2%), and it was incorrectly graded with CDC.

Mitroupolus et al. underlined that special attention should also be paid to proper use of the CDC because it has not been designed/validated to grade intraoperative complications, and any modifications and revisions can be confusing [8]. Recently, Biyani et al. published a new EAU guideline to report and grade intraoperative complications [19]. More recently, Cacciamani et al. proposed an EAU guideline to report intraoperative complications called ICARUS criteria [9]. In the current study, intraoperative complications were graded with Biyani et al. proposal (EAUiaiC). After that, we created a table to show full compatibility with ICARUS criteria. To our knowledge, this is the first time in the literature to publish intraoperative complications with full compliance with ICARUS criteria.

Surgical techniques for RC procedures or routines in postoperative care may cause differences, especially in the number and distribution of conservatively followed complications that do not require surgery. In our clinic, routine broad-spectrum antibiotic prophylaxis, use of antiemetics and bowel movement enhancers, active lung exercises, use of bronchodilators for the first 3 days, early mobilization, and low-molecular-weight heparin prophylaxis are performed for all patients. In our current study, the overall rate for any complication is 69.1%. GIS (27.5%) complications were the most frequent. Most severe postoperative complications requiring intervention (*CDC* ≥ grade IIIb) were found in 18.4%, compatible with the literature [1–3, 21, 22].

In the current study, SIRI, NAC, *BMI* ≥ 25 kg/m^2^, and ACCI were found to be independent predictors of most severe complications. Ornaghi et al. investigated the impact of preoperative nutritional factors (BMI, hypoalbuminemia, and sarcopenia) on complication and mortality rates after RC in a systematic review. They found that high BMI, hypoalbuminemia, and sarcopenia significantly increased the complication rates after RC [23]. Similarly, in the current study, *BMI* ≥ 25 kg/m^2^ was found to be a strong predictor of both 30-day and 90-day complications. We found that patients receiving NAC had fewer severe 90-day complication rates. This may be attributed to the fact that receiving NAC improves surgery-related outcomes. It is well known that NAC does not increase RC complications [24–28]. Additionally, Hoeh et al. reported complications in a detailed manner, and they found that patients who received NAC had significantly less surgical site, cardiac, pulmonary, and genitourinary complications than those who did not [26]. Similarly, Jerlström et al. found that RC patients who received NAC had significantly less GIS complications [27].

Vetterlein et al. found that ACCI and delta hemoglobin were independent predictors of most severe complications [2]. Hirobe et al. found that BMI ≥ 25 kg/m^2^, smoking history, and CCI ≥ 2 were independent risk factors for high-grade complications [22]. Zareba et al. reported that increased number of postoperative complications were observed with increasing ACCI [21]. We also found that *ACCI* ≥ 6 was a significant cutoff to predict most severe complications.

Previously, AGR, PLR, and PNI were found to be associated with oncological outcomes of RC [11, 12, 18]. In the current study, these markers were evaluated to predict complications, and no significant relationship was found. Recently, Claps et al. proposed another immune-nutritional marker called as CONUT score in a multi-institutional retrospective study. They were calculated the CONUT scores according to the serum albumin, lymphocyte count, and cholesterol levels of the patients. They found that high CONUT score (≥ 3) was independently predictive for both complications and oncologic outcomes [29]. Standard methods should be used in studies reporting complications [8]. This principle is the main topic of the current study. Very interestingly, Claps et al. did not clarify how they obtained and reported all complications from five different European centers in a standardized way. They also did not comment or report any limitation about this. Therefore, external validation is obviously needed for their notable results.

SIRI was found to be an independent predictor of 90-day most severe complications. In 2016, Qi et al. first described that SIRI showed good prognostic value in patients with pancreatic cancer [14]. Subsequently, SIRI has also been shown to be associated with various cancers [13, 15–17]. Urbanowicz et al. found SIRI to be associated with post-bypass mortality [30], Jin Z. et al. with ischemic stroke [31], and Lee L. E. et al. with all-cause mortality of vasculitis [32]. The precise mechanisms underlying the association between increasing SIRI levels and poor prognosis prevail to be uncovered. The high level of monocyte and neutrophil count represents a higher tumor burden as they play major roles on increasing the tumor cell migration, invasion, and angiogenesis and suppression of anti-tumor immune reaction [33, 34]. In contrast, lymphocytes have a vital function in anti-tumor defense via direct and antigen-dependent cytotoxic cell death and suppression of the tumor proliferation functions [35]. Therefore, higher level of SIRI indicates increased levels of the immunosuppressive monocytes and neutrophils and decreased levels of the immunogenic lymphocytes. Patients with high tumor burden may be more frail and prone for postoperative complications. In the current study, SIRI was found significantly higher in patients with pT4 stage and/or positive surgical margin. This might be responsible to some extent for severe complications in RC patients.

To our knowledge, first time in the literature, we found that SIRI is independent predictor for severe complications. However, very similar terminology of inflammatory markers causes confusion. For example, the marker obtained by multiplying the neutrophil-to-lymphocyte ratio with the platelet count was named systemic inflammatory index (SII) [36]. Despite that in the current study, SIRI obtained by multiplying neutrophil-to-lymphocyte ratio with monocytes count. Monocytes and platelets have very different and mostly unrelated functions in the human body. On the other hand, Ni et al. were using same terminology (SIRI) for hemoglobin × monocytes/lymphocytes [17], and they analyzed this marker for the cystectomy complications. Again, this study has very different methodology from the current study.

There are some important limitations of this study. Retrospective design of the study is the most important, although Andersen et al. showed that prospectively and retrospectively collected complication data were similar [4]. The current study analyzed the intraoperative complications of open RC. We reviewed the surgical records to investigate iAEs. In laparoscopic or robot-assisted surgery, retrospectively viewing surgical video data can provide additional advantage. Therefore, some EAUiaiC grade 0 complications may not have been mentioned in the surgical records of open RC. Another important limitation is caused from the nature of the CDC. For example, open wounds can be sutured under local anesthesia. However, it may also be performed under general anesthesia just because of patient preference; in this case, the complication grade increases to CDC ≥ IIIb. On the other hand, after the patient leaves the hospital, minor complications may be under-collected. There is also a possibility that complications may be over-looked (not recorded) because patient who experience complications after discharge may administer to local hospitals close to their homes. However, almost all of the patients are referred to our tertiary referral center due to the general health practice in our country. EAU muscle-invasive bladder cancer guideline noted that higher RC hospital volume is associated with lower postoperative mortality rates and higher quality of care [37]. And they also recommended performing at least 10, and preferably > 20, RCs per hospital/per year. Despite the fact that this study meets the minimum criteria, more is better. Cohorts with more cases could improve complications. On the other hand, a guideline was published on the use of the ERAS protocol in RC in late 2013 [38]. The fact that our study included patients before this date and also our ERAS compliance was 22% limits our results. This study was designed to assess all RCs performed by high- and low-volume surgeons and fellowship trained surgeons, and no case was excluded based on surgeon case volume alone. However, all RCs were performed under the supervision of a senior surgeon (OD).

## Conclusions

In the current study, for the first time in the literature, detailed open RC complications were reported within a standardized manner, concordant with both the ICARUS and Martin criteria and EAU guideline recommendations. RC is surgery that has high complication rates. Both intraoperative and postoperative complications obviously increased with using standard methodology. ACCI, SIRI, *BMI* ≥ 25 kg/m^2^, and the absence of NAC were independent predictive factors for most severe complications (*CDC* ≥ IIIb). The cutoff values of > 2.35 for *SIRI* > 4 for ACCI could be predict of *CDC* ≥ grade IIIb complications.

## Supplementary Information

**Additional file 1: Supplementary Fig. 1.** Flowchart illustrating the recruiting process.

**Additional file 2: Supplementary Table 1.** Re-operations and Re-admissions causes of patients.

**Additional file 3: Supplementary Table 2.** The fulfilment of EAU quality criteria.

## Data Availability

All of the material is owned by the authors, and/or no permissions are required.
