# Supplementary figures and images for: Reporting perioperative complications of radical cystectomy: the influence of using standard methodology based on ICARUS and EAU quality criteria
Source: World J Surg Oncol. 2023 Feb 23;21:58. doi: 10.1186/s12957-023-02943-9 (PMC9948374; doi:10.1186/s12957-023-02943-9)

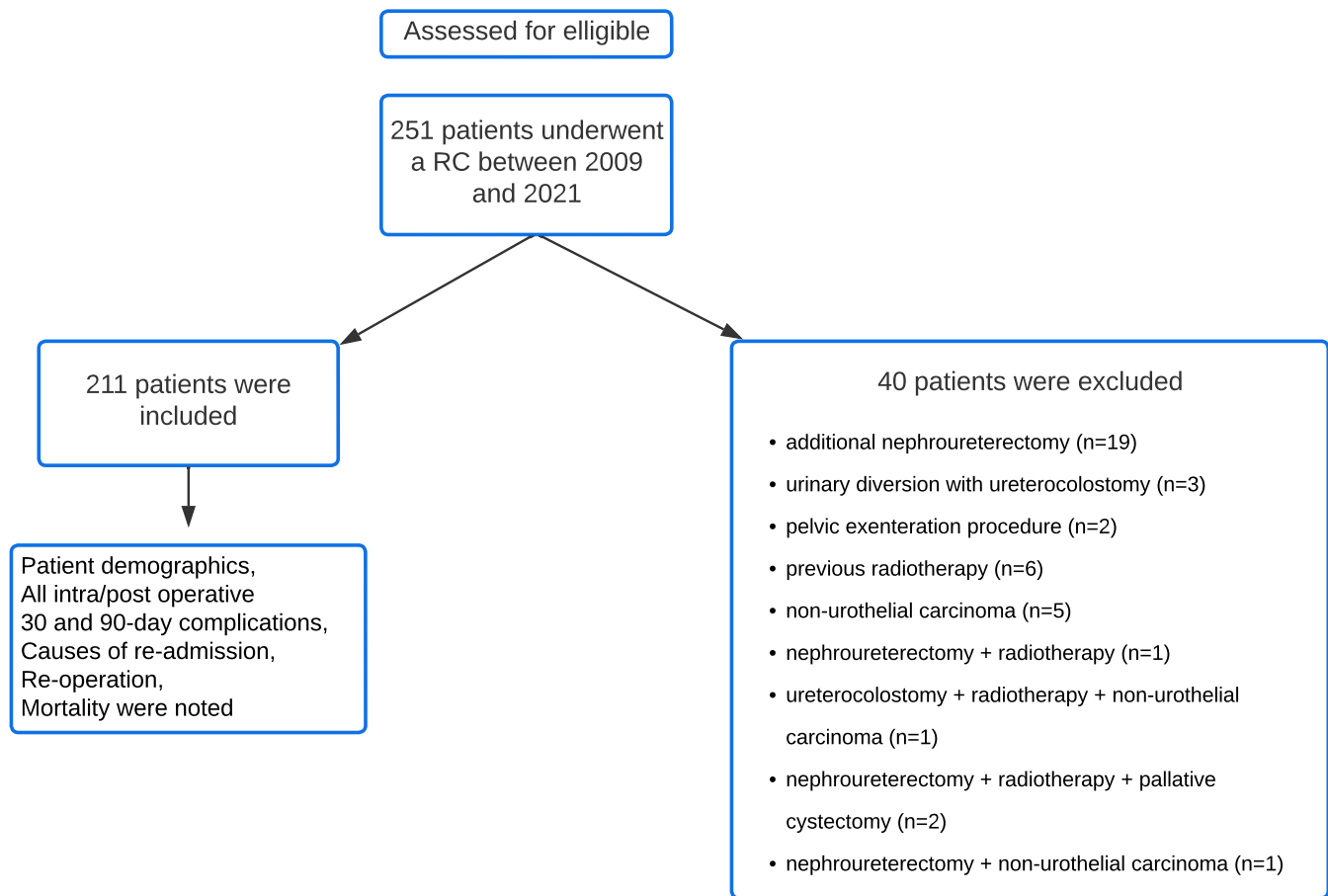

Supplement: Supplementary file 1 — Additional file 1: Supplementary Fig. 1. Flowchart illustrating the recruiting process. [file 12957_2023_2943_MOESM1_ESM.pdf]
