# Supplementary material for: Reporting perioperative complications of radical cystectomy: the influence of using standard methodology based on ICARUS and EAU quality criteria
Source: World J Surg Oncol. 2023 Feb 23;21:58. doi: 10.1186/s12957-023-02943-9 (PMC9948374; doi:10.1186/s12957-023-02943-9)
Supplement: Supplementary file 2 — Additional file 2: Supplementary Table 1. Re-operations and Re-admissions causes of patients. [file 12957_2023_2943_MOESM2_ESM.docx]

| **Supplementary Table 1.** *Re-operations and Re-admissions causes of patients.* | | | |
| --- | --- | --- | --- |
| **Re-operations, n (%)** | | **30-days** | **90-days** |
| Gastrointestinal operations | | 13 (41.9) | 20 (50) |
|  | Colostomy | 1 (3.2) | 2 (5) |
|  | Ileum resection | 3 (9.6) | 5 (12.5) |
|  | Ileostomy | 2 (6.4) | 5 (12.5) |
|  | Ileum perforation repair | 2 (6.4) | 3 (7.5) |
|  | Ileal re-anastomosis | 4 (12.9) | 4 (10) |
|  | Cholecystectomy | 1 (3.2) | 1 (2.5) |
| Evisceration | | 12 (38.7) | 12 (30) |
| Incisional hernia | | 4 (12.9) | 4 (10) |
| Ureteral re-implantation | | 1 (3.2) | 1 (2.5) |
| Abscess drainage | | 1 (3.2) | 3 (7.5) |
| Total | | 31 (100) | 40 (100) |
|  | |  |  |
| **Re-admissions, n (%)** | | **30-days** | **90-days** |
| Gastrointestinal | | 19 (54.2) | 23 (51.1) |
| Infectious | | 3 (8.5) | 7 (15.5) |
| Wound | | 4 (11.4) | 4 (8.8) |
| Genitourinary | | 3 (8.5) | 4 (8.8) |
| Cardiac | | 0 (0) | 1 (2.2) |
| Pulmonary | | 1 (2.8) | 1 (2.2) |
| Thromboembolic | | 1 (2.8) | 1 (2.2) |
| Neurological | | 2 (5.7) | 2 (4.4) |
| Prolonged lymphatic drainage | | 2 (5.7) | 2 (4.4) |
| Total | | 35 (100) | 45 (100) |
|  | | | |
