# Supplementary material for: Reporting perioperative complications of radical cystectomy: the influence of using standard methodology based on ICARUS and EAU quality criteria
Source: World J Surg Oncol. 2023 Feb 23;21:58. doi: 10.1186/s12957-023-02943-9 (PMC9948374; doi:10.1186/s12957-023-02943-9)
Supplement: Supplementary file 3 — Additional file 3: Supplementary Table 2. The fulfilment of EAU quality criteria. [file 12957_2023_2943_MOESM3_ESM.docx]

| *Supplementary Table 2. The fulfilment of EAU quality criteria.* | |
| --- | --- |
| **EAU quality Criteria** | **Compliance** |
| 1. Define the method of accruing data | Retrospective digitalized chart review of our medical records for inpatient, outpatient and the emergency clinics |
| 2. Define who collected the data | Two urology residents (BC, KC) |
| 3. Indicate the duration of follow-up | 30-day and 90-day after open radical cystectomy |
| 4. Include outpatient information | Outpatient information was included |
| 5. Include mortality data and causes of death | Mortality and causes of death were reported in table 2 |
| 6. Include definitions of complications | All complications were defined |
| 7. Define procedure-specific complications | Procedure-specific complications were defined |
| 8. Report intraoperative and postoperative complications separately | We separately reported intraoperative and postoperative complications |
| 9. Use a severity grading system for postoperative complications  (avoiding the distinction minor/major); Clavien-Dindo system is  recommended | The Clavien-Dindo classification and the Comprehensive Complication Index were used |
| 10. Postoperative complications should be presented in a table either by  grade or by complication type (specific grades should always be  provided; grouping is not accepted) | A detailed table of postoperative complications, including grading, treatment, frequencies, and proportions was provided |
| 11. Include risk factors | The age-adjusted Charlson comorbidity index was included into analyses |
| 12. Include readmissions and causes | A detailed table of readmissions ( and causes) and reoperations (types and causes)were provided |
| 13. Include reoperations, types and causes |  |
| 14. Include the percentage of patients lost to follow-up | Follow-up was available for all patients, given the retrospective review of digitalized  charts and 30-day and 90-day follow-up period |
